# Evidence of horizontal transmission of *Wolbachia w*Ccep in rice moths parasitized by *Trichogramma chilonis* and its persistence across generations

**DOI:** 10.3389/finsc.2024.1519986

**Published:** 2024-12-09

**Authors:** C. T. Lai, Y. T. Hsiao, Li-Hsin Wu

**Affiliations:** Department of Plant Medicine, National Pingtung University of Science and Technology, Neipu, Pingtung, Taiwan

**Keywords:** *Wolbachia* acquisition, symbiosis, *Wolbachia* persistence, biological control, climate impact

## Abstract

The horizontal transmission of endosymbionts between hosts and parasitoids plays a crucial role in biological control, yet its mechanisms remain poorly understood. This study investigates the dynamics of horizontal transfer of *Wolbachia* (*w*Ccep) from the rice moth, *Corcyra cephalonica*, to its parasitoid, *Trichogramma chilonis*. Through PCR detection and phylogenetic analysis, we demonstrated the presence of identical *w*Ccep strains in both host and parasitoid populations, providing evidence for natural horizontal transmission. To investigate thoroughly, *Wolbachia*-free colonies were acquired through tetracycline treatment, and the initial density of *w*Ccep in host eggs significantly influences transmission efficiency. High-density *w*Ccep infections led to rapid transmission, with F1 parasitoid titers increasing by as much as 100-fold, while low-density infections exhibited more gradual increases. Additionally, without continuous exposure to infected hosts, *w*Ccep density in *T. chilonis* diminished over generations. These findings enhance our understanding of *Wolbachia*’s transfer dynamics and have important implications for developing effective and sustainable biological control strategies using parasitoid wasps, particularly in managing *Wolbachia*-related pest populations in agricultural systems.

## 1 Introduction

*Wolbachia*, an extensively studied endosymbiotic bacterium present across diverse arthropod taxa, has emerged as a crucial focus in biological control research due to its capacity to manipulate host reproduction. This maternally inherited endosymbiont is estimated to infect over 50% of insect species (1). Although *Wolbachia* primarily spreads through vertical transmission from mother to offspring, accumulating evidence indicates that horizontal transfer—the acquisition of *Wolbachia* by a novel host from an unrelated donor—occurs with greater frequency than previously recognized across phylogenetically diverse insect taxa. (2).

*Wolbachia* was first discovered in the mosquito *Culex pipiens* (3). Within insect hosts, *Wolbachia* is primarily found in reproductive tissues but can also be present in somatic cells and tissues like salivary glands, hemolymph, and the gut (4). To increase its prevalence within host populations, *Wolbachia* manipulates host reproduction through mechanisms such as inducing parthenogenesis, feminization, male-killing, and cytoplasmic incompatibility (CI), which confers a reproductive advantage to infected females (5). Beyond reproductive manipulations, *Wolbachia*’s potential to enhance host fitness is a fascinating area of research, as it can protect against pathogens like dengue virus (6) and West Nile virus (7) and influence mitochondrial DNA variation (8), prompting interest in its use for vector-borne disease control (9, 10).

Horizontal transfer events have been documented in various insect orders, including Diptera (11), Hymenoptera (12–14), Lepidoptera (15, 16), Araneae (17), and Hemiptera (18). These events have been observed within and across different species, facilitated by various mechanisms such as parasitism, predation, and shared ecological niches (19). The potential for horizontal transfer is further supported by the discovery of highly similar *Wolbachia* strains in distantly related insect species, suggesting possible shifts between host lineages (15). Additionally, novel *Wolbachia* strains have been identified as inducing reproductive incompatibility in previously uninfected hosts, as observed in whiteflies (16). Furthermore, research on parasitoid wasps has demonstrated the horizontal transfer of parthenogenesis-inducing *Wolbachia* in laboratory settings (20), highlighting the adaptability and transferability of *Wolbachia* across different insect species.

Intriguingly, studies have reported highly similar *Wolbachia* strains (>95% sequence similarity) infecting phylogenetically distant butterfly species from the families Papilionidae and Nymphalidae (2). Likewise, ant species have been found harboring identical *Wolbachia* strains to their kleptoparasitic ant cricket hosts, indicating potential horizontal transfer events (19). Frydman et al. (21) demonstrated that *Wolbachia* could migrate from the hemolymph to reproductive tissues in *Drosophila melanogaster* following microinjection, reaching the oocytes within 15 days. These findings highlight the ability of *Wolbachia* to spread across species boundaries.

While horizontal transfer events have been documented, the factors governing the successful establishment and persistence of acquired *Wolbachia* strains within novel hosts remain poorly understood. Sanaei et al. (22) proposed a four-stage model for successful horizontal transfer: (1) contact and entry into the new host, (2) survival and practical replication within the new host, (3) efficient vertical transmission within the new host lineage, and (4) spread and maintenance within the new host population. Each stage presents unique challenges, including overcoming the host’s immune response, adapting to the new cellular environment, and ensuring vertical transmission and long-term persistence. Previous molecular evidence has shown that *T. chilonis* and other *Trichogramma* species collected in Taiwan harbor the same *Wolbachia* strain (*w*Pip) as their factitious host, the rice moth (23, 24).

In this context, the current study explores the possibility of horizontal transfer of *Wolbachia* between the rice moth and its parasitoid, the *Trichogramma* wasp. To validate the dynamics of this potential horizontal transfer, rice moth eggs with different titers of *w*Ccep were provided to *T. chilonis*. To assess the successful acquisition and persistence of *w*Ccep in *T. chilonis* across generations. The current study contributes to the understanding of *Wolbachia* horizontal transfer dynamics, elucidating the factors that facilitate or impede the successful acquisition and maintenance of novel *Wolbachia* strains within new hosts.

## 2 Method

### 2.1 Collection of insect sources and rearing conditions

Egg masses of *Ostrinia furnacalis* (Asian corn borer) parasitized by *Trichogramma* wasps were collected from corn fields in Yanpu Township, Pingtung, Taiwan, in July-August 2020. The collected egg masses were placed in a growth chamber at 25°C. One day after adult emergence, individual female rice moths were isolated and reared on eggs of the factitious host, rice moth, *Corcyra cephalonica*, in 50 ml centrifuge tubes at 25°C, 20 ± 5% RH, and 12:12 h (light: dark) photoperiod to establish the laboratory population of the parasitoid, *Trichogramma chilonis*.

### 2.2 PCR detection

To confirm the infection status, ten female *T. chilonis* from each iso-female line were collected, and their genomic DNA was extracted using the ALS Tissue Genomic DNA Extraction Kit. PCR amplification of *Wolbachia wsp* and *ftsZ* genes was performed using wsp81F/691R and FtsZBf/Br primers, respectively. The primers amplify the *Trichogramma* COI gene as an internal reference (5, 25, 26). The PCR reaction mixture consisted of 2 μL of template DNA, 0.5 μL of each forward and reverse primer, 4 μL of FIREPol^®^ MasterMix, and 13 μL of ddH2O. The PCR reactions were performed under the following conditions: initial denaturation at 95°C for 2 minutes, followed by 35 cycles of 95°C for 30 seconds, 55°C for 1 minute, and 72°C for 1 minute, with a final extension at 72°C for 7 minutes. The same protocols were also used to determine the *Wolbachia* infection rate of the laboratory population of *C. cephalonica*.

### 2.3 Phylogenetic analysis of *Wolbachia*, *w*Ccep

The *wsp* and *ftsZ* gene sequences of *Wolbachia* from the iso-female lines and the laboratory population of *C. cephalonica* were analyzed using maximum likelihood estimation (MLE) with 1,000 bootstrap replicates (33), and compared with *Wolbachia* strains in the NCBI database.

### 2.4 Establishing *Wolbachia*, *w*Ccep-free (*w*Ccep-) *C. cephalonica* and *T. chilonis* populations

*C. cephalonica* was reared on rice bran treated with 4.8 mg/g tetracycline under conditions of 30°C, 20 ± 5% RH, and 12L:12D photoperiod. After the fifth generation, *Wolbachia* absence in the *C. cephalonica* population was confirmed by qPCR. *T. chilonis* were reared on *C. cephalonica* eggs free of *w*Ccep. Their *Wolbachia* status was monitored by qPCR each generation to establish a *w*Ccep- *T. chilonis* population. To minimize the potential effects of tetracycline residues, the antibiotic-treated *C. cephalonica* population was reared on untreated rice bran for at least one generation before being used in subsequent experiments.

### 2.5 Evaluation of horizontal transfer of *Wolbachia*, *w*Ccep in *T. chilonis*

#### 2.5.1 Quantity of *Wolbachia*, *w*Ccep in T. chilonis

With the 49th generation of *T. chilonis* reared on *w*Ccep- hosts, *w*Ccep density in parasitoids was monitored by qPCR for 15 generations to determine if *w*Ccep could persist without infected hosts. Each generation was tested, and each sample was run in triplicate. *T. chilonis* individuals with *w*Ccep were used as the control group. The qPCR reaction mixture consisted of 5 μL iQ SYBR Green Supermix (Bio-Rad), 0.25 μL of each primer, 2.5 μL ddH_2_O, and 2 μL extracted DNA, for a total volume of 10 μL. The target gene, *wsp*, was detected using the qWspcc5R and qWspcc5F primers, while the reference gene, *COI*, was detected using the qTcCOI5R and qTcCOI5F primers (**Supplementary Table 1**). The qPCR conditions were 95°C for 3 minutes, followed by 40 cycles of 95°C for 10 seconds and 59.5°C for 30 seconds.

The density of *Wolbachia*, *w*Ccep, was determined using the ΔΔCt method. First, by subtracting the Ct value of the reference gene from the Ct value of the target *Wolbachia*, *w*Ccep gene, the ΔCt value was calculated for each sample. Then, the ΔΔCt value was calculated by subtracting the ΔCt of the control group from the ΔCt of the treatment group. Finally, the fold change in *w*Ccep density was determined using the formula: Fold gene expression = 2^-(ΔΔCt)^.

#### 2.5.2 Effect of *Wolbachia*, *w*Ccep density on horizontal transmission efficiency

After 15 generations of rearing *T. chilonis* on *w*Ccep- hosts, they were then provided with either (1) *wCcep*-infected *C. cephalonica* eggs (high *Wolbachia* titer treatment) or (2) tetracycline-treated (2.4 mg/g) *C. cephalonica* eggs (low *Wolbachia* titer treatment). To compare the differences, qPCR at each generation monitored the density and replication rate of *w*Ccep in the parasitoids.

#### 2.5.3 DNA extraction and quantification of *Wolbachia*, *w*Ccep density in *C. cephalonica*

Eggs were collected from two populations of *C. cephalonica*: one infected with *w*Ccep and one treated with antibiotics for five generations. To extract DNA from *C. cephalonica* eggs, they were crushed with a pestle in 1.5 mL microcentrifuge tubes. Then 50 μL of 5% Chelex solution and 1 μL of Proteinase K solution were added, and the samples were incubated at 56°C for 40 minutes, followed by incubation at 95°C for 10 minutes.

Each generation was tested with twenty samples, each run in triplicate. The qPCR reaction mixture was composed of 5 μL of iQ SYBR Green Supermix (Bio-Rad), 0.25 μL of each primer, 2.5 μL of ddH_2_O, and 2 μL of extracted DNA, for a total volume of 10 μL. The primers qWspcc5R and qWspcc5F were utilized to detect the target *w*Ccep product, while the qCcCOI1R and qCcCoI1F primers were used for the reference gene (refer to **Supplementary Table 1**). Statistical analyses were performed using R software (version 4.3.0; 27). Kruskal-Wallis tests followed by *post-hoc* comparisons using the Benjamini-Hochberg correction to control for multiple testing and maintain the false discovery rate at 0.05.

## 3 Results

### 3.1 Phylogenetic analysis of *Wolbachia*, *w*Ccep

**Figure 1:**
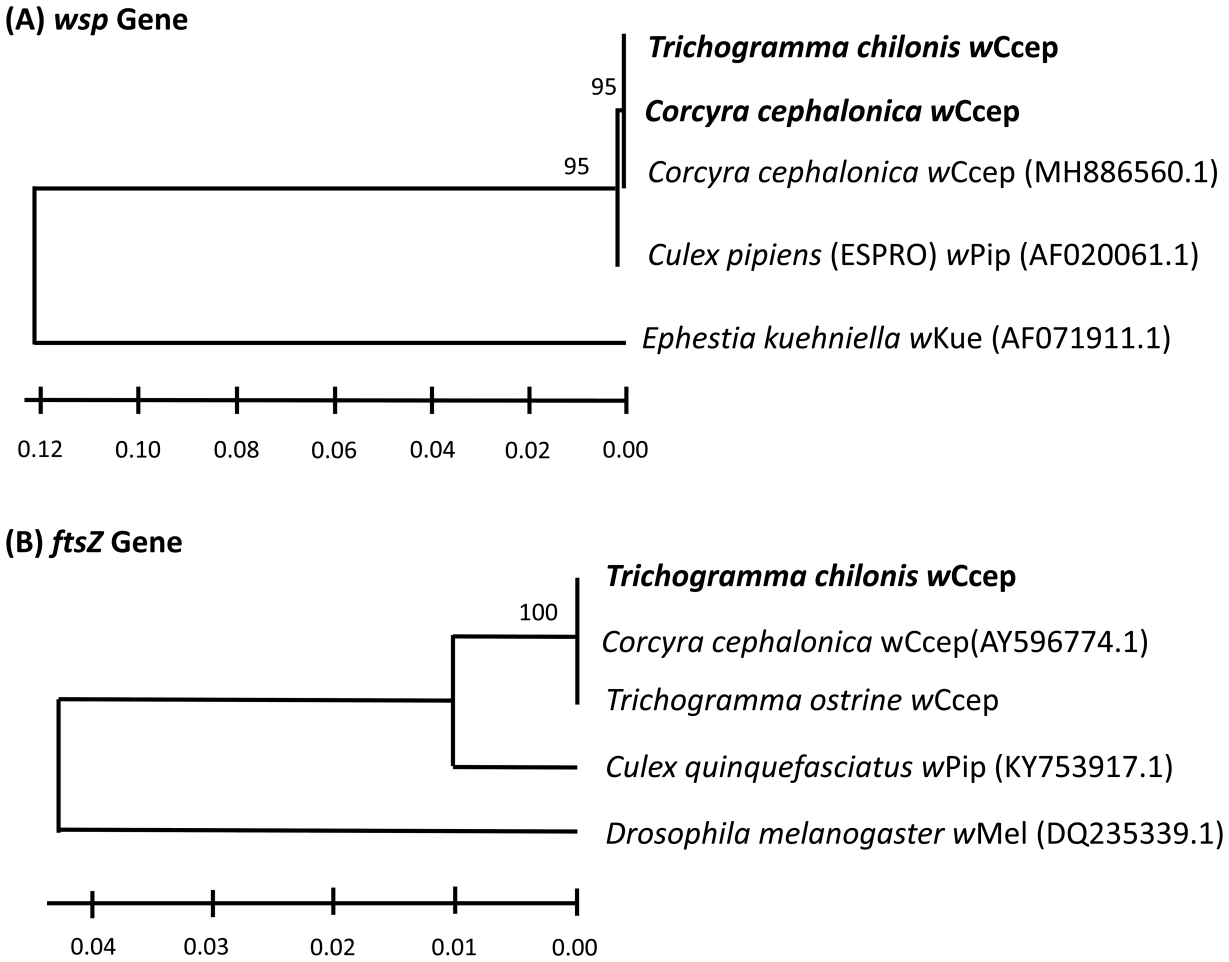
Neighbor-joining tree for *Wolbachia* strains based on partial sequences of **(A)** *wsp* gene and **(B)** *ftsZ* gene. Distances were calculated using the Maximum Likelihood Estimation in MEGA X (33). Bootstrap support values (1000 replicates). Bold text indicates the laboratory populations tested in this study. *Wolbachia* strains are identified by the host species from which they were isolated, followed by an NCBI accession number.

After discovering stable *Wolbachia* infection in *T. chilonis* from Yanpu, Pingtung, phylogenetic analyses confirmed that the *Wolbachia* strain detected in our *T. chilonis* iso-female lines belongs to supergroup B, specifically the *w*Ccep strain, identical to the strain found in their laboratory host, *C. cephalonica*. This suggests that the *w*Ccep detected in *T. chilonis* was likely transferred from the laboratory-maintained *C. cephalonica* population. When comparing the *wsp* gene fragments, there were no differences between the *w*Pip infection recorded in 2016 and *w*Ccep, with only a 1% divergence detected in the *ftsZ* gene fragment (**Figure 1**).

### 3.2 Establishing *Wolbachia*, *w*Ccep-free (*w*Ccep-) *C. cephalonica* and *T. chilonis* populations

**Figure 2:**
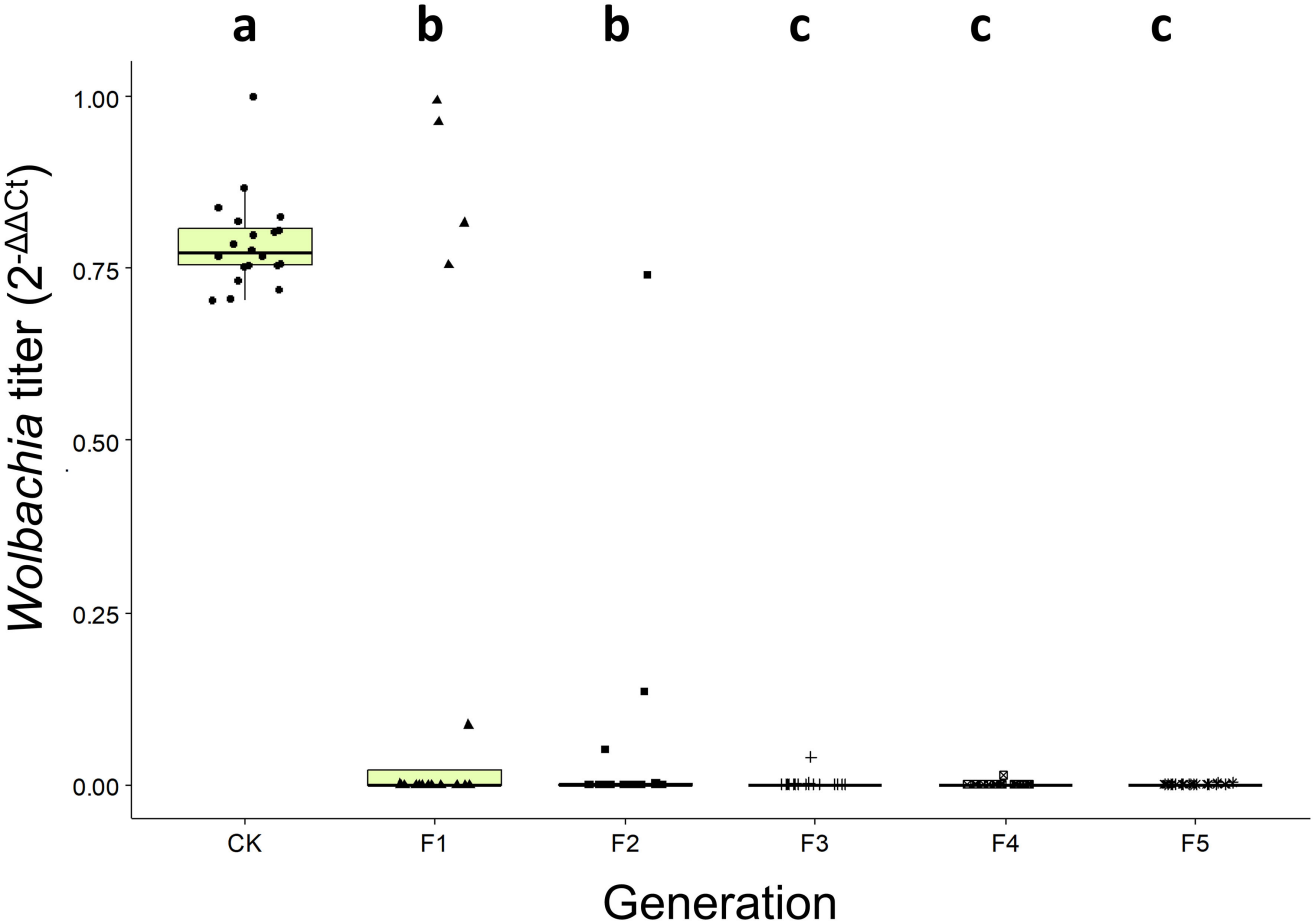
*Wolbachia* (*w*Ccep) titer on *C. cephalonica* after 1 to 5 generations of tetracycline treatment. Different letters indicate significant differences (*p* < 0.05; Kruskal-Wallis test with *post-hoc* Benjamini-Hochberg test).

Analysis revealed significant variation in *w*Ccep density across five generations of *C. cephalonica* maintained on tetracycline-supplemented artificial diet (4.8 mg/g) (Kruskal-Wallis chi-squared = 63.613, d.f = 5, *p* = 2.173 × 10^12^). As treatment generations increased, *Wolbachia* density consistently decreased compared to the infected population. A significant decline in *w*Ccep density was observed from the first generation and continued to decrease in subsequent generations. By the fifth generation, *w*Ccep was no longer detectable, indicating that continuous tetracycline treatment for five generations effectively eradicated *w*Ccep infection in *C. cephalonica*. This procedure successfully established a *Wolbachia*-free (*w*Ccep-) strain of *C. cephalonica* (**Figure 2**).

**Figure 3:**
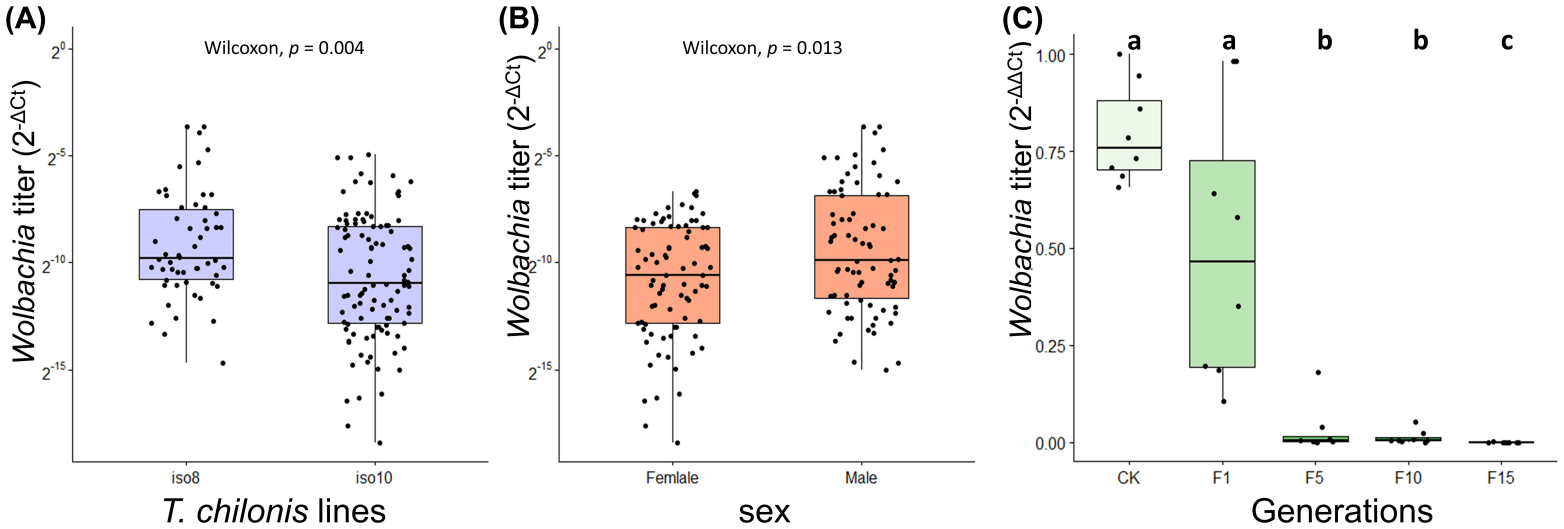
*Wolbachia* (*w*Ccep) titer on **(A)** *T. chilonis* iso-female lines, **(B)** male and female *T. chilonis*, and **(C)** *T. chilonis* parasitizing *Wolbachia*-free *C. cephalonica* across 15 generations. Different letters indicate significant differences (*p* < 0.05; Kruskal-Wallis test with *post-hoc* Benjamini-Hochberg correction).

Further, qPCR of *w*Ccep titers in *T. chilonis* iso-female lines revealed significant variations among different iso-female strains; male individuals exhibited significantly higher *w*Ccep titers than females (**Figures 3A, B**). In a laboratory-maintained population of *T. chilonis* reared for 49 generations, parasitizing *w*Ccep-free *C. cephalonica* eggs led to a gradual decrease in *w*Ccep density as parasitism generations increased (F1-F15) (Kruskal-Wallis chi-squared = 32.908, d.f = 4, *p* = 1.248 × 10^-6^). The decline in *w*Ccep density within *T. chilonis* was observed starting from the first generation. These findings suggest that without additional supplementation of *w*Ccep, the bacterial density in *T. chilonis* progressively diminishes, indicating the necessity of continuous parasitism of *C. cephalonica* to maintain *w*Ccep levels (**Figure 3C**).

**Figure 4:**
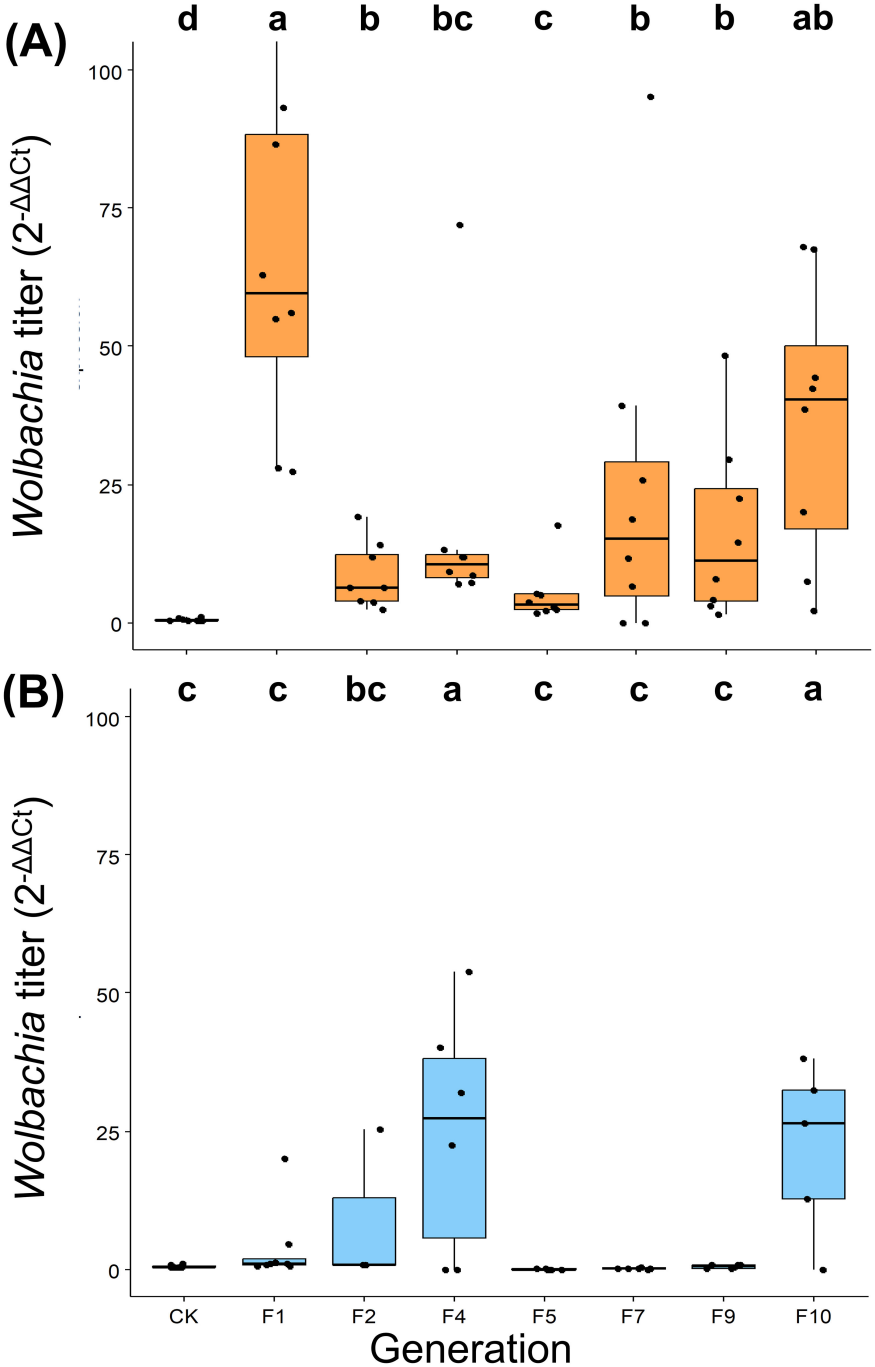
*Trichogramma chilonis* re-infected with *Wolbachia* (*w*Ccep) by parasitizing *C. cephalonica* with **(A)** high *w*Ccep titer (2^-ΔΔCt^ = 1) and **(B)** low *w*Ccep titer (2^-ΔΔCt^ = 0.014). The letters indicate significant differences (*p*<0.05; Kruskal-Wallis test with *post-hoc* Benjamini and Hochberg test).

Our investigation into *w*Ccep transmission dynamics revealed significant titer variations depending on the initial infection levels in host eggs. The F15 generation of *T. chilonis*, parasitizing high-density *w*Ccep eggs (2^-ΔΔCt^ = 1), showed dramatic titer increases, with some F1 individuals exhibiting nearly 100-fold higher densities compared to controls (Kruskal-Wallis chi-squared = 36.43, d.f = 7, *p* = 6.013 × 10^-6^). In contrast, parasitization of tetracycline-treated, low-density *w*Ccep eggs (2^-ΔΔCt^ = 0.014) resulted in a slower, more gradual increase in *w*Ccep titers (Kruskal-Wallis chi-squared = 22.757, d.f = 7, *p* = 1.879 × 10^-3^). Notably, both scenarios demonstrated significant titer fluctuations across generations F1 to F10, with the high-density treatment showing more pronounced variability, notably a marked decrease after F1 followed by a resurgence around F7 (**Figures 4A, B**).

## 4 Discussion

This study demonstrated that *Wolbachia* strain *w*Ccepundergoes horizontal transmition from *C. cephalonica* to uninfected *T. chilonis* through host-parasitoid interactions. This transmission occurred and led to successful proliferation within the new host. The 100% sequence similarity in the *ftsZ* gene between *w*Ccep strains from field-collected *C. cephalonica* and *T. chilonis* in southwestern Taiwan. The comparison of existing *wsp* and *ftsZ* sequences in the PubMLST database also showed that the *Wolbachia* strain isolated from *T. chilonis* is closely related to the *wCcep* strain within the STC-41 clonal complex, which is usually found in Lepidoptera hosts (2) (**Supplementary Table 2**). These results are consistent with previous studies that have shown *Wolbachia* horizontal transmission in *Trichogramma* species, such as intraspecific transfer in *T. kaykai* through superparasitism (28) and interspecific transmission of *w*Den from *T. dendrolimi* to *T. evanescens* through microinjection (29).

Importantly, our study demonstrates that the initial density of *w*Ccep in host eggs affects the timing and efficiency of horizontal transmission. Higher *w*Ccep densities facilitated more rapid transmission, aligning with other findings. For example, Liu et al. (30) showed that increased *Wolbachia* inoculation frequency in *Drosophila melanogaster* led to higher infection densities. Meanwhile, Toomey et al. (31) identified high *Wolbachia* density as a critical factor for horizontal transmission in *D. melanogaster*. These collective findings suggest that the initial encounter density of *Wolbachia* is crucial not only for successful horizontal transmission but also for subsequent proliferation and vertical transmission to offspring in new hosts.

Notably, following the parasitization of *w*Ccep-infected *C. cephalonica* eggs by uninfected *T. chilonis*, we observed a rapid initial increase in *w*Ccep density in the offspring generation. However, this density did not consistently increase over subsequent generations; it exhibited significant fluctuations. We hypothesize that these generational density fluctuations may be attributed to the host’s innate immune response to the newly acquired *Wolbachia*, leading to unstable *w*Ccep densities in the novel host (32).

Hu and Li (16) reported a case where *w*Ccep successfully induced reproductive incompatibility in whiteflies after infection via microinjection. *w*Ccep-infected *C. cephalonica* populations exhibited similar characteristics, suggesting its potential to induce cytoplasmic incompatibility in *T. chilonis* (unpublished data). Additionally, *Wolbachia* infection may affect reproduction and fitness, which will be further investigated in future studies.

## 5 Conclusions

The current study provides evidence through molecular analysis and re-infection trials demonstrating that *Wolbachia* (*wCcep*) can be transmitted from *C. cephalonica* to *T. chilonis*. We established a *w*Ccep-free *C. cephalonica* colony over five generations using tetracycline. The transmission timing depends on population density, and *w*Ccep can persist in *T. chilonis* for one to two generations. These findings are important for biological control programs using *T. chilonis* and managing *Wolbachia* infections in mass-rearing systems.

## Data Availability

The datasets presented in this study can be found in online repositories. The names of the repository/repositories and accession number(s) can be found in the article/**Supplementary Material**.
