# Supplementary material for: Evidence of horizontal transmission of Wolbachia wCcep in rice moths parasitized by Trichogramma chilonis and its persistence across generations
Source: Front Insect Sci. 2024 Dec 9;4:1519986. doi: 10.3389/finsc.2024.1519986 (PMC11663936; doi:10.3389/finsc.2024.1519986)
Supplement: Supplementary file 1 [file Table1.docx]

Supplementary Material

# Supplementary Data

Supplementary Material should be uploaded separately on submission. Please include any supplementary data, figures and/or tables.

Supplementary material is not typeset so please ensure that all information is clearly presented, the appropriate caption is included in the file and not in the manuscript, and that the style conforms to the rest of the article.

# Supplementary Figures and Tables

**Supplementary Table S1.** List of qPCR primers used in this study. The primers were designed using NCBI Primer-BLAST. Accession No. refers to the sequence ID in the NCBI database used as the template for primer design.

| Primer | Primer sequence (5’-3’) | Gene | Product size(bp) | Accession NO. |
| --- | --- | --- | --- | --- |
| qWspCc5F | TGGCAGCATTTTCAGGATTG | *wsp* | 176 | MH886560.1 |
| qWspCc5R | AACACCAGCTTTTGCTTGAT |  |  |  |
| qTcCOI2F | ACCCGATATAGCATTCCCTC | *COI* | 162 | MK611830.1 |
| qTcCOI2R | ACTGAAGGACCCCTATGAGA |  |  |  |
| qCcCOI1F | GCTCCTGATATAGCTTTCCCT |  | 121 | MK611832.1 |
| qCcCOI1R | ACACAGTTCATCCAGTTCCT |  |  |  |

**Supplementary Table S2. List of *Wolbachia* isolates retrieved from the PubMLST database based on *wsp* typing results.**

| id | strain | host | | gatB | | coxA | | hcpA | | ftsZ | | fbpA | | ST | | clonal complex | | wsp | | HVR1 | | HVR2 | | HVR3 | | HVR4 | |
| --- | --- | --- | --- | --- | --- | --- | --- | --- | --- | --- | --- | --- | --- | --- | --- | --- | --- | --- | --- | --- | --- | --- | --- | --- | --- | --- | --- |
| - | **Current study** | ***Trichogramma chilonis*** | | **-** | | **-** | | **-** | | **36** | | **-** | | **-** | |  | | **10** | | **10** | | **8** | | **10** | | **8,238** | |
| 29 | Cpip_B | *Culex pipiens* | | 4 | | 3 | | 3 | | 22 | | 4 | | 9 | |  | | 10 | | 10 | | 8 | | 10 | | 8 | |
| 30 | Cqui_B | *Culex pipiens* | | 4 | | 3 | | 3 | | 22 | | 4 | | 9 | |  | | 10 | | 10 | | 8 | | 10 | | 8 | |
| 40 | Hbol_B_wBol1 | *Hypolimnas bolina* | | 4 | | 14 | | 40 | | 73 | | 4 | | 125 | |  | | 10 | | 10 | | 8 | | 10 | | 8 | |
| 101 | Nang_B | *Nacaduba angusta* | | 39 | | 14 | | 40 | | 36 | | 4 | | 41 | | STC-41 | | 10 | | 10 | | 8 | | 10 | | 8 | |
| 128 | Amir_B | *Azanus mirza* | 39 | | 14 | | 40 | | 36 | | 4 | | 41 | | STC-41 | | 10 | | 10 | | 8 | | 10 | | 8 | |  |
| 131 | Carg_B | *Celastrina argiolus* | 39 | | 14 | | 40 | | 36 | | 4 | | 41 | | STC-41 | | 10 | | 10 | | 8 | | 10 | | 8 | |  |
| 195 | Eman_B_CI | *Eurema mandarina* | 39 | | 14 | | 40 | | 36 | | 4 | | 41 | | STC-41 | | 10 | | 10 | | 8 | | 10 | | 8 | |  |
| 196 | Eman_B_CI | *Eurema mandarina* | 39 | | 14 | | 40 | | 36 | | 4 | | 41 | | STC-41 | | 10 | | 10 | | 8 | | 10 | | 8 | |  |
| 197 | Eman_B_CI | *Eurema mandarina* | 39 | | 14 | | 40 | | 36 | | 4 | | 41 | | STC-41 | | 10 | | 10 | | 8 | | 10 | | 8 | |  |
| 198 | Eman_B_CI | *Eurema mandarina* | 39 | | 14 | | 40 | | 36 | | 4 | | 41 | | STC-41 | | 10 | | 10 | | 8 | | 10 | | 8 | |  |
| 199 | Eman_B_CI | *Eurema mandarina* | 39 | | 14 | | 40 | | 36 | | 4 | | 41 | | STC-41 | | 10 | | 10 | | 8 | | 10 | | 8 | |  |
| 210 | Cama_B_wAma | *Colotis amata* | 16 | | 14 | | 40 | | 36 | | 4 | | 150 | | STC-41 | | 10 | | 10 | | 8 | | 10 | | 8 | |  |
| 212 | Cner_B_wNer | *Cepora nerissa* | 4 | | 14 | | 3 | | 36 | | 4 | | 145 | | STC-41 | | 10 | | 10 | | 8 | | 10 | | 8 | |  |
| 213 | Cpom_B_wPom | *Catopsilia pomona* | 39 | | 14 | |  | | 36 | |  | |  | |  | | 10 | | 10 | | 8 | | 10 | | 8 | |  |
| 215 | Cros_B_wRos | *Castalius rosimon* | 9 | |  | | 40 | |  | |  | |  | |  | | 10 | | 10 | | 8 | | 10 | | 8 | |  |
| 218 | Deuc_B_wEuc | *Delias eucharis* | 39 | | 14 | | 40 | | 36 | | 4 | | 41 | | STC-41 | | 10 | | 10 | | 8 | | 10 | | 8 | |  |
| 219 | Ehec_B_wHec | *Eurema hecabe* | 102 | | 14 | | 29 | | 36 | | 42 | | 157 | | STC-41 | | 10 | | 10 | | 8 | | 10 | | 8 | |  |
| 220 | Ehec_B_wHec | *Eurema hecabe* | 39 | | 14 | | 40 | | 36 | | 4 | | 41 | | STC-41 | | 10 | | 10 | | 8 | | 10 | | 8 | |  |
| 221 | Ehec_B_wHec | *Eurema hecabe* | 39 | | 14 | | 40 | | 36 | | 4 | | 41 | | STC-41 | | 10 | | 10 | | 8 | | 10 | | 8 | |  |
| 222 | Ehec_B_wHec | *Eurema hecabe* | 39 | | 14 | | 40 | | 36 | | 4 | | 41 | | STC-41 | | 10 | | 10 | | 8 | | 10 | | 8 | |  |
| 225 | Hbol_B_wBol | *Hypolimnas bolina* | 9 | | 14 | | 40 | | 73 | | 4 | | 148 | | STC-41 | | 10 | | 10 | | 8 | | 10 | | 8 | |  |
| 228 | Jiph_B_wIph | *Junonia iphita* | 9 | |  | | 100 | | 73 | | 4 | |  | |  | | 10 | | 10 | | 8 | | 10 | | 8 | |  |
| 229 | Jlem_B_wLem | *Junonia lemnonias* | 4 | | 14 | | 40 | | 36 | | 4 | | 146 | | STC-41 | | 10 | | 10 | | 8 | | 10 | | 8 | |  |
| 230 | lep_B_F21 | *Ixias pyrene* | 39 | | 14 | | 40 | | 36 | | 4 | | 41 | | STC-41 | | 10 | | 10 | | 8 | | 10 | | 8 | |  |
| 233 | lep_B_F49 | *Udaspes folus* | 39 | |  | |  | | 36 | | 4 | |  | |  | | 10 | | 10 | | 8 | | 10 | | 8 | |  |
| 234 | lep_B_F65 | *Zizeeria knysna* | 39 | | 14 | | 40 | | 36 | | 4 | | 41 | | STC-41 | | 10 | | 10 | | 8 | | 10 | | 8 | |  |
| 235 | Lnin_B_wNin | *Leptosia nina* | 39 | | 14 | | 40 | | 7 | | 4 | | 152 | | STC-41 | | 10 | | 10 | | 8 | | 10 | | 8 | |  |
| 239 | Pdem_B_wDem | *Papilio demoleus* | 39 | |  | |  | |  | |  | |  | |  | | 10 | | 10 | | 8 | | 10 | | 8 | |  |
| 240 | Pmah_B_wMah | *Pseudozizeeria maha* | 39 | | 14 | | 40 | | 36 | | 4 | | 41 | | STC-41 | | 10 | | 10 | | 8 | | 10 | | 8 | |  |
| 241 | Pmah_B_wMah | *Pseudozizeeria maha* | 39 | | 14 | | 40 | | 36 | | 4 | | 41 | | STC-41 | | 10 | | 10 | | 8 | | 10 | | 8 | |  |
| 246 | Tnys_B_wNys | *Telicada nyseus* | 4 | | 14 | | 40 | | 36 | | 4 | | 146 | | STC-41 | | 10 | | 10 | | 8 | | 10 | | 8 | |  |
| 247 | Tnys_B_wNys | *Telicada nyseus* | 4 | | 14 | | 40 | | 73 | | 4 | | 125 | |  | | 10 | | 10 | | 8 | | 10 | | 8 | |  |
| 248 | Tnys_B_wNys | *Telicada nyseus* | 4 | |  | | 40 | |  | |  | |  | |  | | 10 | | 10 | | 8 | | 10 | | 8 | |  |
| 249 | Yast_B_wAst | *Ypthima asterope* | 39 | |  | |  | | 36 | |  | |  | |  | | 10 | | 10 | | 8 | | 10 | | 8 | |  |
| 270 | wBol_B_wBol1 | *Hypolimnas bolina* | 4 | | 14 | | 40 | | 73 | | 4 | | 125 | |  | | 10 | | 10 | | 8 | | 10 | | 8 | |  |
| 297 | Ehec_B_CI | *Eurema hecabe* | 39 | | 14 | | 40 | | 36 | | 4 | | 41 | | STC-41 | | 10 | | 10 | | 8 | | 10 | | 8 | |  |
| 457 | Carg_B | *Celastrina argiolus* | 16 | | 14 | | 40 | | 36 | | 4 | | 150 | | STC-41 | | 10 | | 10 | | 8 | | 10 | | 8 | |  |
| 458 | Cosc_B | *Clossiana oscarus* | 39 | | 14 | | 40 | | 36 | | 4 | | 41 | | STC-41 | | 10 | | 10 | | 8 | | 10 | | 8 | |  |
| 459 | Earg_B | *Everes argiades* | 39 | | 14 | | 40 | | 36 | | 4 | | 41 | | STC-41 | | 10 | | 10 | | 8 | | 10 | |  | |  |
| 460 | Ealc_B | *Everes alcetas* | 16 | | 14 | | 40 | | 36 | | 4 | | 150 | | STC-41 | | 10 | | 10 | | 8 | | 10 | | 8 | |  |
| 466 | Olut_B | *Opistograptis luteolata* | 16 | | 14 | | 40 | | 36 | | 4 | | 150 | | STC-41 | | 10 | | 10 | | 8 | | 10 | | 8 | |  |
| 467 | Pama_B | *Polyommatus amandus* | 39 | | 14 | | 40 | | 36 | | 4 | | 41 | | STC-41 | | 10 | | 10 | | 8 | | 10 | | 8 | |  |
| 473 | Tbet_B | *Thecla betulae* | 39 | | 14 | | 40 | | 36 | | 4 | | 41 | | STC-41 | | 10 | | 10 | | 8 | | 10 | | 238 | |  |
| 649 | Cfag_B_7H.1AK | *Cydia fagiglandana* | 16 | | 14 | | 40 | | 36 | | 4 | | 150 | | STC-41 | | 10 | | 10 | | 8 | | 10 | | 8 | |  |
| 650 | Cfag_B_6A.1AK | *Cydia fagiglandana* | 16 | | 14 | | 40 | | 36 | | 4 | | 150 | | STC-41 | | 10 | | 10 | | 8 | | 10 | | 8 | |  |
| 651 | Cfag_B_5E.1AK | *Cydia fagiglandana* | 16 | | 14 | | 40 | | 36 | | 4 | | 150 | | STC-41 | | 10 | | 10 | | 8 | | 10 | | 8 | |  |
| 652 | Cfag_B_2C.1AK | *Cydia fagiglandana* | 16 | | 14 | | 40 | | 36 | | 4 | | 150 | | STC-41 | | 10 | | 10 | | 8 | | 10 | | 8 | |  |
| 653 | Cspl_B_8E.1BK | *Cydia splendana* | 16 | | 14 | | 40 | | 36 | | 4 | | 150 | | STC-41 | | 10 | | 10 | | 8 | | 10 | | 8 | |  |
| **1631** | **Ccep_B_BJ** | ***Corcyra cephalonica*** | **39** | | **14** | | **40** | | **36** | | **4** | | **41** | | **STC-41** | | **10** | | **10** | | **8** | | **10** | | **8** | |  |
| 1669 | wJuv | *Leptidea juvernica* | 39 | | 14 | | 40 | | 36 | | 4 | | 41 | | STC-41 | | 10 | | 10 | | 8 | | 10 | | 8 | |  |
| 1670 | wSin2 | *Leptidea sinapis* | 101 | | 14 | | 40 | | 7 | | 89 | | 466 | |  | | 10 | | 10 | | 8 | | 10 | | 8 | |  |
| 1672 | wAmu | *Leptidea amurensis* | 39 | | 14 | | 40 | | 36 | | 4 | | 41 | | STC-41 | | 10 | | 10 | | 8 | | 10 | | 8 | |  |
| 1808 | wPip | *Culex quinquefasciatus* | 4 | | 3 | | 3 | | 22 | | 4 | | 9 | |  | | 10 | | 10 | | 8 | | 10 | | 8 | |  |
| 2039 | JHB | *Culex quinquefasciatus* | 4 | | 3 | | 3 | | 22 | | 4 | | 9 | |  | | 10 | | 10 | | 8 | | 10 | | 8,238 | |  |
| 2043 | wPip | *Culex quinquefasciatus* | 4 | | 3 | | 3 | | 22 | | 4 | | 9 | |  | | 10 | | 10 | | 8 | | 10 | | 8,238 | |  |
| 2050 | Ob_Wba | *Operophtera brumata* | 39 | | 14 | |  | | 36 | | 4 | |  | |  | | 10 | | 10 | | 8 | | 10 | | 8,238 | |  |
| 2067 | wPip_Mol | *Culex molestus* | 4 | | 3 | | 3 | | 22 | | 4 | | 9 | |  | | 10 | | 10 | | 8 | | 10 | | 8,238 | |  |
| **2127** | **SYLI2103** | ***Corcyra cephalonica*** | **39** | | **14** | | **40** | | **36** | | **4** | | **41** | | **STC-41** | | **10** | | **10** | | **8** | | **10** | | **8,238** | |  |
